# Downregulation of type 3 inositol (1,4,5)-trisphosphate receptor decreases breast cancer cell migration through an oscillatory Ca^2+^ signal

**DOI:** 10.18632/oncotarget.20327

**Published:** 2017-08-18

**Authors:** Abdallah Mound, Alexia Vautrin-Glabik, Arthur Foulon, Béatrice Botia, Frédéric Hague, Jan B. Parys, Halima Ouadid-Ahidouch, Lise Rodat-Despoix

**Affiliations:** ^1^ Laboratory of Cellular and Molecular Physiology (EA-4667), “Ion Channels in Breast Cancer”, SFR CAP-SANTE (FED-4231), University of Amiens, UFR Sciences, 80039 Amiens, France; ^2^ Laboratory of Molecular and Cellular Signalling, Department of Cellular and Molecular Medicine, Campus Gasthuisberg O/N1- bus 802-K U Leuven, B-3000 Leuven, Belgium

**Keywords:** breast cancer, migration, type 3 inositol 1,4,5-trisphosphate receptor, Ca^2+^

## Abstract

Breast cancer remains a research priority due to its invasive phenotype. Although the role of ion channels in cancer is now well established, the role of inositol (1,4,5)-trisphosphate (IP_3_) receptors (IP_3_Rs) remains enigmatic. If the three IP_3_Rs subtypes expression have been identified in various cancers, little is known about their physiological role. Here, we investigated the involvement of IP_3_R type 3 (IP_3_R_3_) in the migration processes of three human breast cancer cell lines showing different migration velocities: the low-migrating MCF-7 and the highly migrating and invasive MDA-MB-231 and MDA-MB-435S cell lines. We show that a higher IP_3_R3 expression level, but not IP_3_R1 nor IP_3_R2, is correlated to a stronger cell line migration capacity and a sustained calcium signal. Interestingly, silencing of IP_3_R3 highlights an oscillating calcium signaling profile and leads to a significant decrease of cell migration capacities of the three breast cancer cell lines. Conversely, stable overexpression of IP_3_R3 in MCF-7 cells significantly increases their migration capacities. This effect is completely reversed by IP_3_R3 silencing. In conclusion, we demonstrate that IP_3_R3 expression level increases the migration capacity of human breast cancer cells by changing the calcium signature.

## INTRODUCTION

Most frequently occurring cancer in women, breast cancer also presents the highest death rate. Cancer mortality is not correlated to tumor growth (due to proliferation mechanisms) but, in 90% of cases, to formation of metastasis [1] by migrating and invading cells. One of the mechanisms behind this invasion and metastasis process in breast cancer is the epithelial–mesenchymal transition (EMT) that allows epithelial cancer cells to dedifferentiate and undergo the rear-to-front polarization and to acquire high migratory capacity, invasiveness, enhanced resistance to apoptosis, and stem cell properties [2, 3]. Thus, EMT provides an explanation for why epithelial cancers with poor differentiation status are generally more aggressive and prone to metastasize than more differentiated cancers [4, 5]. Cell migration is a complex multistep process that involves protrusions of the leading edge of the cell, formation of adhesion complexes and the release of adhesions at the cell rear. Calcium (Ca^2+^) is a key effector of these migratory mechanisms by modulating the focal adhesion turnover or the cytoskeletal organization [6]. Ca^2+^ increases can occur in the form of waves, spikes or oscillations with various impact on cell migration progression [7]. Several plasma membrane channels that increase Ca^2+^ into the cytosol, such as the transient receptor potential (TRP) channels [8] and Orai/STIM channels [9, 10] have been described in cancer cell migration, but implication of inositol-(1,4,5)-trisphosphate (IP_3_) receptors (IP_3_Rs) [11] and ryanodine receptors (RyRs) [12] in such process remain fragmented.

IP_3_R protein subtypes (IP_3_R1, IP_3_R2 and IP_3_R3) are encoded by three different genes in mammals, however the resulting proteins share high similarity in their primary sequences and are expressed to varying degrees in different cell types [13]. Interestingly, Miyakawa *et al*. [14] demonstrated that IP_3_Rs subtypes differ by a specific Ca^2+^ signature, which is associated to various sensitivity to endogenous modulators such as IP_3_, Ca^2+^ and ATP. Thus, activation of IP_3_R1 generates very rapidly damped Ca^2+^ oscillations, IP_3_R2 stimulation produces regular and robust Ca^2+^ oscillations, whereas IP_3_R3 functions as an anti-Ca^2+^ oscillatory units with a Ca^2+^ transient signature [14, 15] and is able to modulate the spatiotemporal pattern of intracellular Ca^2+^ signals induced by ATP [16, 17].

Overexpression of IP_3_Rs has been demonstrated in various cancer types where a pro-apoptotic [18, 19] and pro-invasive [20, 21] roles of IP_3_Rs have been established. Concerning IP_3_R3 subtype, it appears as a key actor of carcinogenesis as its expression level is correlated with colorectal carcinoma aggressiveness [22], whereas its inhibition reduces breast cancer cell proliferation [17], migration, invasion and survival of glioblastoma cells [20]. Furthermore, we recently demonstrated that IP_3_R3 co-localizes and interacts, both at molecular and functional levels, with voltage- and Ca^2+^-dependent K^+^ channels (BK_Ca_). This interaction appears to specifically occur in cancerous cells and increases cancer cell proliferation [23]. Based on our previous works [17, 23], we investigated the role of IP_3_R3 dependent Ca^2+^ signaling in the migratory process of three human breast cancer cell lines with different migration capacities. Our results clearly show that silencing of IP_3_R3 (siR3) reveals an oscillating Ca^2+^ signature and significantly decreases migration of invasive breast cancer cells (MDA-MB-231 and MDA-MB-435S). Conversely, IP_3_R3 overexpression in MCF-7 cells modifies the ATP-induced Ca^2+^ response from an oscillatory into a sustained signal and significantly increases their migration capability. This effect is completely reversed by siR3. Thus, our results strengthen the involvement of IP_3_R3 as a key player in the migration of breast cancer cells through modulation of their Ca^2+^ signature.

## RESULTS

### IP_3_R3 expression level is specifically correlated to migration capacity of breast cancer cell lines

**Figure 1:**
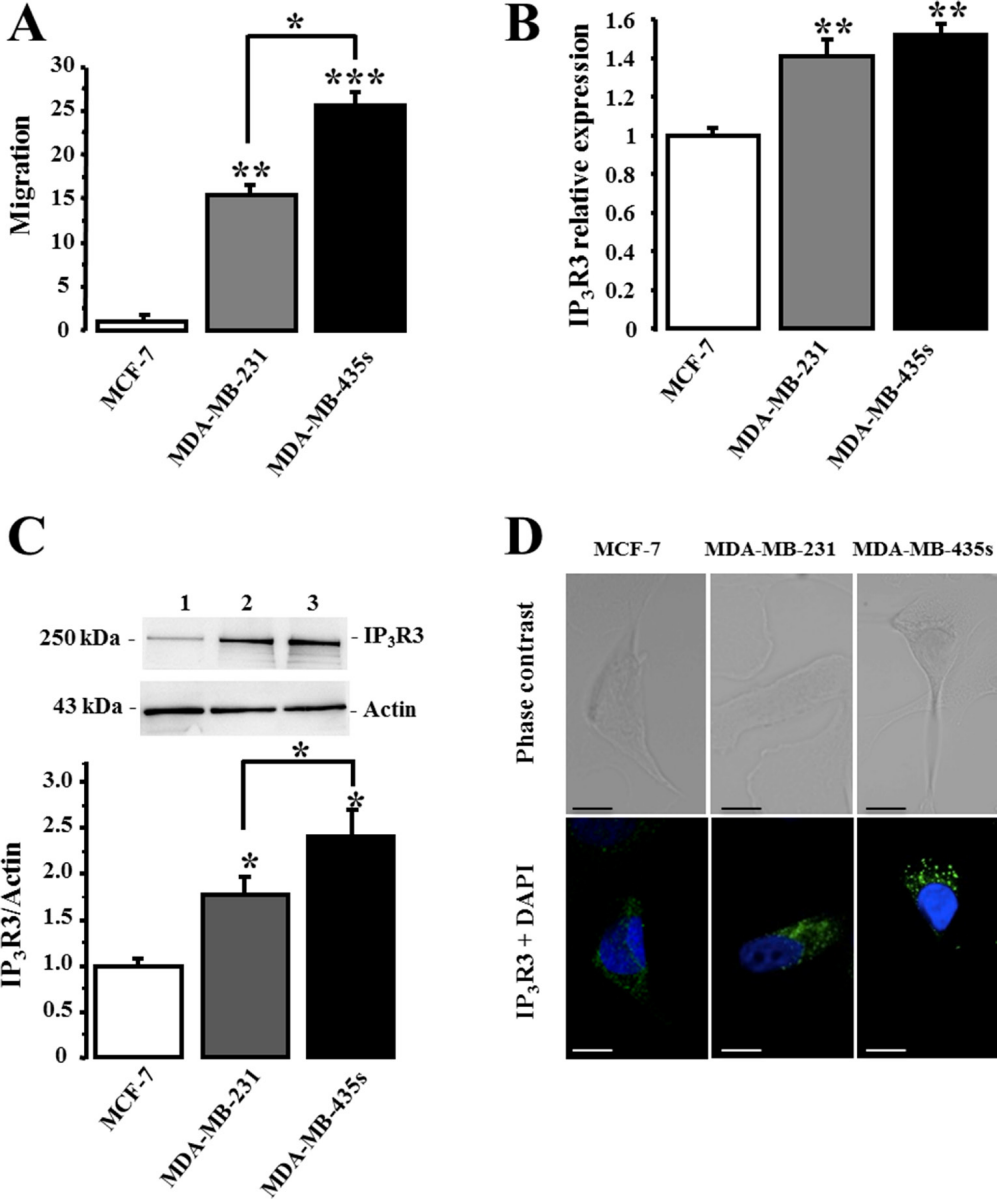
IP_3_R3 expression level is correlated to migration capacity of breast cancer cell lines (**A**) Relative migration capacities of MCF-7, MDA-MB-231 and MDA-MB-435S breast cancer cell lines were evaluated using Boyden chamber migration assay. For each experiment, the number of migrating cells per area for each condition was normalized to MCF-7 cells. (**B**) IP_3_R3 mRNA relative expression detected in MCF-7, MDA-MB-231 and MDA-MB-435S cells was quantified using RT-qPCR and results are expressed as average ± SEM of IP_3_R3/β-actin mRNA ratio. (**C**) IP_3_R3 protein expression level was analyzed by Western-blot in MCF-7 (1), MDA-MB-231 (2) and MDA-MB-435S cells (3). Actin protein was used as loading control and quantitative analysis are the average of three independent experiments. Histogram summarizes quantification of IP_3_R3 expression level in the three cell lines. (**D**) Immunolabelling of IP_3_R3 in breast cancer cell lines MCF-7 (1), MDA-MB-231 (2) and MDA-MB-435S (3). Cells were immunostained with anti-IP_3_R3 antibody (green) and nuclei were stained with DAPI (blue). Scale bar = 20 μm. Values are reported as mean ± SEM normalized to the MCF-7 cells (*N* = 3). *p < 0.05, **p < 0.01, ***p < 0.001.

**Figure 2:**
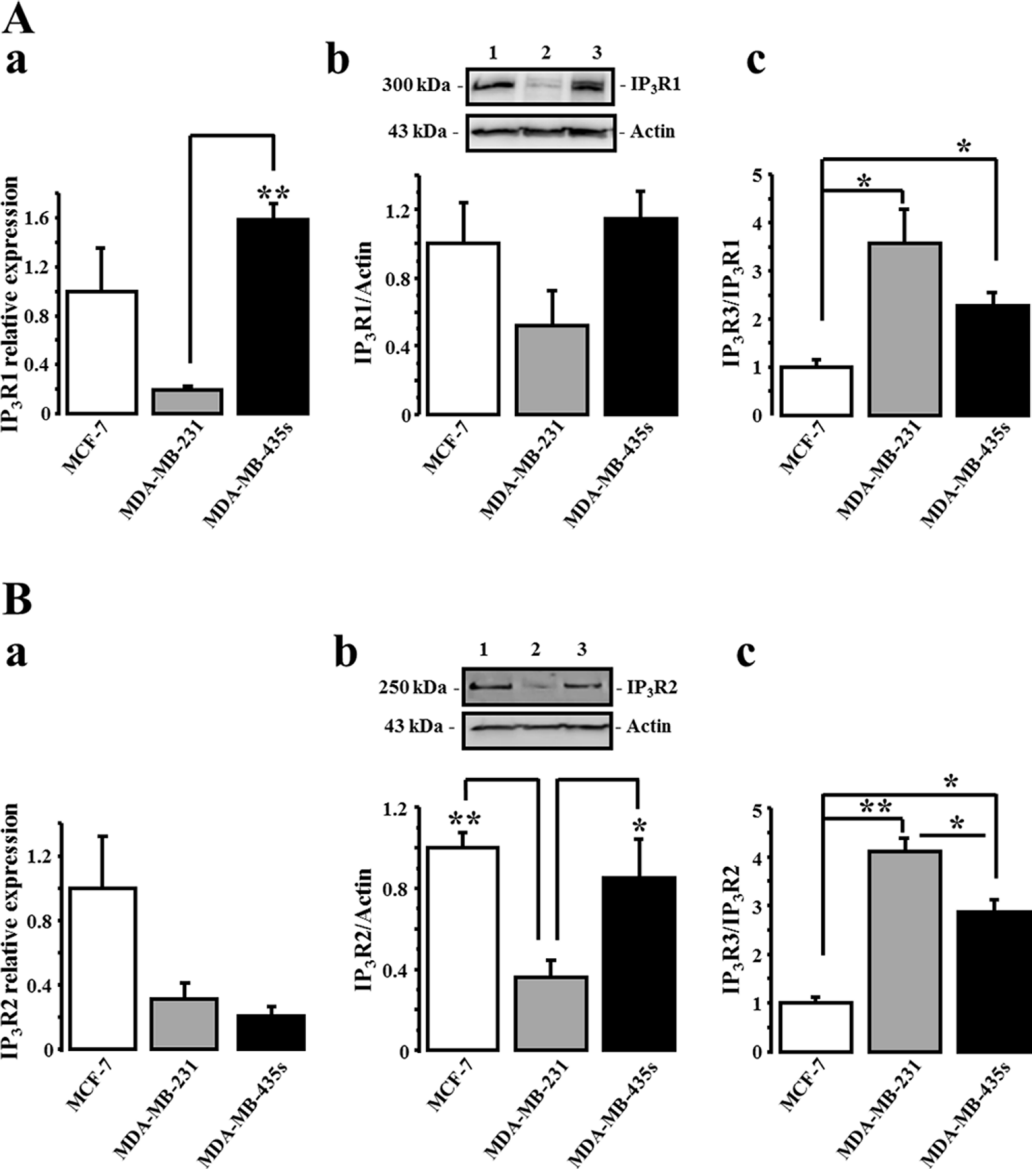
The expression level of IP_3_R1 and IP_3_R2 is independent of breast cancer migration capacity IP_3_R1 (**A**) and IP_3_R2 (**B**) transcripts (a) and protein levels (b) were quantified in MCF-7 (1), MDA-MB-231 (2) and MDA-MB-435S (3) cells. IP_3_R3 protein expression level was rationalized to IP_3_R1 (Ac) and to IP_3_R2 (Bc) expression levels. Actin protein was used as loading control and, quantitative analyses of Western-blots are the average of three independent experiments. Values are reported as mean ± SEM normalized to the MCF-7 cells (*N* = 3). *p < 0.05, **p < 0.01.

We have previously reported that IP_3_R3 is the unique isoform to positively regulate the 17-beta estradiol-induced proliferation of the estrogen-dependent MCF-7 cell line [17]. Here, we investigated the expression levels of the three IP_3_Rs subtypes in three breast cancer cell lines with various degree of malignancy: a very low migrating- (MCF-7), a metastatic- (MDA-MB-231), and a highly metastatic (MDA-MB-435S) cell lines. We first realized cell migration measurements using the Boyden transwell chamber assays in order to characterize and compare the migration potential of each cell line. The relative rank order of cell migration capacity of MCF-7, MDA-MB-231 and MDA-MB-435S is reported in Figure 1A. Relative cell migration is 1 ± 0.66, 15.43 ± 1.33 and 25.48 ± 1.59 *N* = 3, for MCF-7, MDA-MB-231 and MDA-MB-435S, cells respectively. In parallel, we measured and compared the expression level of IP_3_R3 at the RNA (Figure 1B) and at the protein (Figure 1C) levels in each cell line. Interestingly, it appears that a higher RNA and protein IP_3_R3 expression level is correlated to a higher migration capacity of breast cancer cell lines. The relative IP_3_R3 RNA and protein expression levels are respectively in MCF-7 (1 ± 0.04 (*N* = 3) and 1 ± 0.06 (*N* = 3)); MDA-MB-231 (1.41 ± 0.08 (*N* = 3, *p* = 0.003) and 1.78 ± 0.18 (*N* = 3, *p* = 0.04)) and MDA-MB-435S (1.52 ± 0.06 (*N* = 3, *p* = 0.004) and 2.41 ± 0.28 (*N* = 3, *p* = 0.02)). Immunostaining with anti-IP_3_R3 antibody confirmed this marked labeling in highly migrating cells MDA-MB-435S compared to MDA-MB-231 and MCF-7 cells (Figure 1D). This correlation between the cell migration potential and the IP_3_R3 expression is specific to IP_3_R3 subtype, since it is not observed with the others IP_3_R1 and IP_3_R2 subtypes (Figure 2). To appreciate the IP_3_R3 expression compared to the two others IP_3_R subtypes, we also investigated RNA and protein expression levels of both IP_3_R1 (Figure 2Aa and 2Ab, Table 1) and IP_3_R2 (Figure 2Ba and 2Bb, Table 1) in the same batch of the three cell lines. Similarly to IP_3_R3, IP_3_R1 is predominantly expressed in MDA-MB435s (Table 1), whereas IP_3_R2 appeared as expressed in MCF-7 as in MDA-MB-435S cell lines (Table 1). Moreover, the rationalization of IP_3_R3 expression to IP_3_R1 (Figure 2Ac) or IP_3_R2 (Figure 2Bc) protein expression levels confirmed its overexpression in migrating cell lines compared to the low migrating MCF-7 cell line. The slight IP_3_R1 and IP_3_R2 levels measured in MDA-MB-231 cells accentuate the predominance of IP_3_R3 in these cells (Figure 2Ac and 2Bc), even if MDA-MB-435S cells show the highest IP_3_R3 expression level (Figure 1B). Taken together, our results establish, for the first time, a specific correlation between IP_3_R3 expression level and the cell migration capacities in breast cancer cell lines.

**Table 1:** RNA and protein expression levels of IP_3_R1 and IP_3_R2 in breast cancer cell lines

Cell type	IP_3_R1		IP_3_R2	
	ARN value	Protein value	ARN value	Protein value
MCF-7	1 ± 0.36	1 ± 0.23	1 ± 0.32	1 ± 0.07
MDA-MB-231	0.19 ± 0.006	0.52 ± 0.19	0.31 ± 0.09	0.36 ± 0.08^b^
MDA-MB-435S	1.58 ± 0.11^a^	1.15 ± 0.15	0.21 ± 0.05	0.85 ± 0.19^c^

The values are mean of triplicate assays (*N* = 3). The significant differences are shown with superscript letters and *p* values are: a = 0.009; b = 0.008 and c = 0.021.

### IP_3_R3 silencing drastically decreases migration of breast cancer cell lines

**Figure 3:**
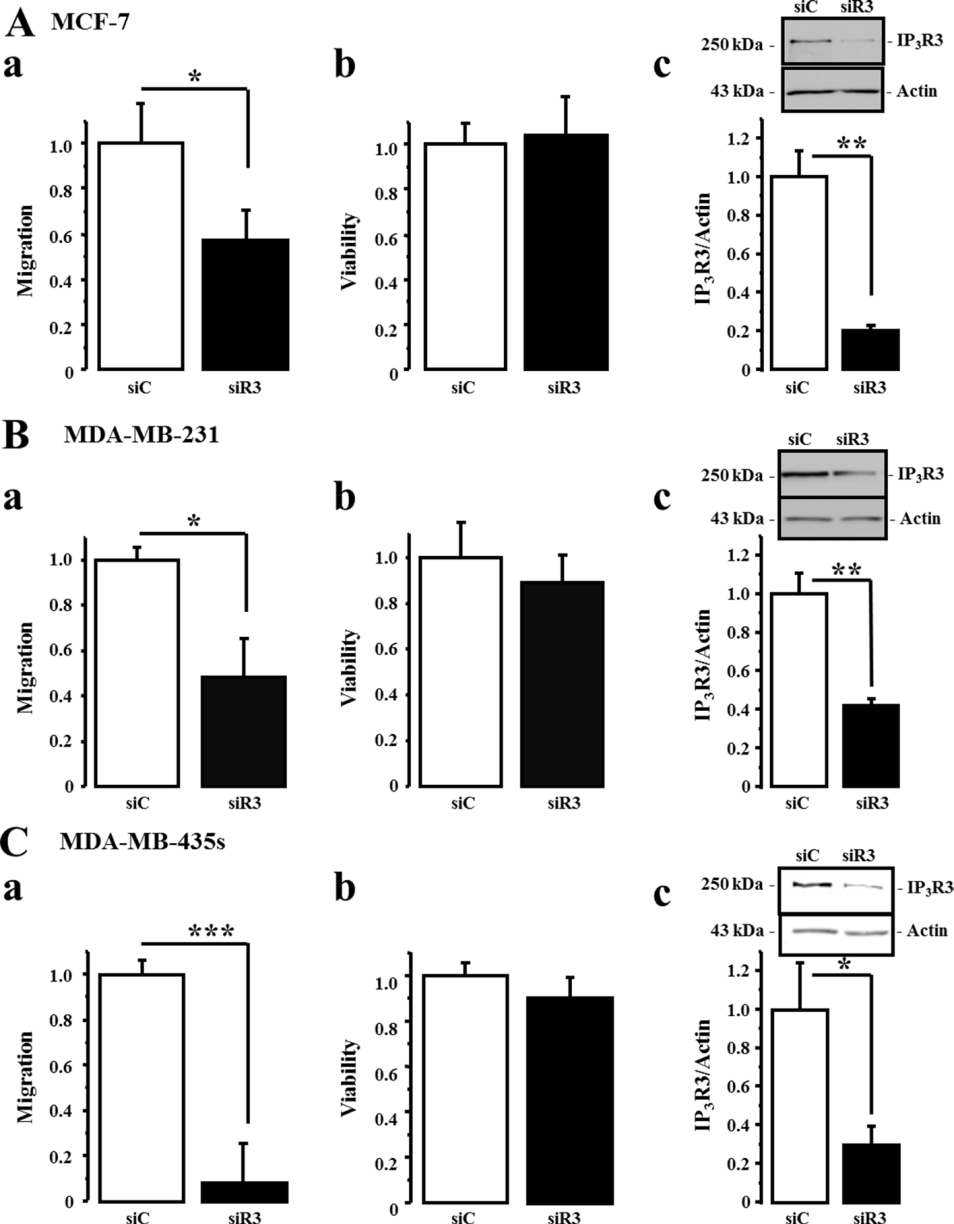
IP_3_R3 gene silencing decreases breast cancer cell migration MCF-7, MDA-MB-231 and MDA-MB-435S breast cancer cell lines were transfected either by a small interfering RNA (siRNA) targeting IP_3_R3 (siR3) or a control siRNA (siC). The migration was then measured at 24 h for MCF-7 and 16 h for MDA-MB-231 and MDA-MB-435S cells, according to their different migration capacities and velocities. Migration tests were performed using Boyden chamber as described above. In all cases, measurements were carried out 72 h after the transfection with respective siRNAs (MCF-7, (**Aa**) MDA-MB-231, (**Ba)** and MDA-MB-435S cells, (**Ca**) compared to control conditions (siC-transfected cells). In the three cell lines, the cell viability was not affected by siR3 (**Ab**, **Bb** and **Cb)** for MCF-7, MDA-MB-231 and MDA-MB-435S cells, respectively). Silencing efficiency on IP_3_R3 protein expression was confirmed by Western-blot experiments on MCF-7, (**Ac**) MDA-MB-231, (**Bc)** and MDA-MB-435S cells, (**Cc**) Values are reported as mean ± SEM normalized to the corresponding cells transfected with control siRNA (siC) (*N* = 3). *p < 0.05, **p < 0.01.

**Figure 4:**
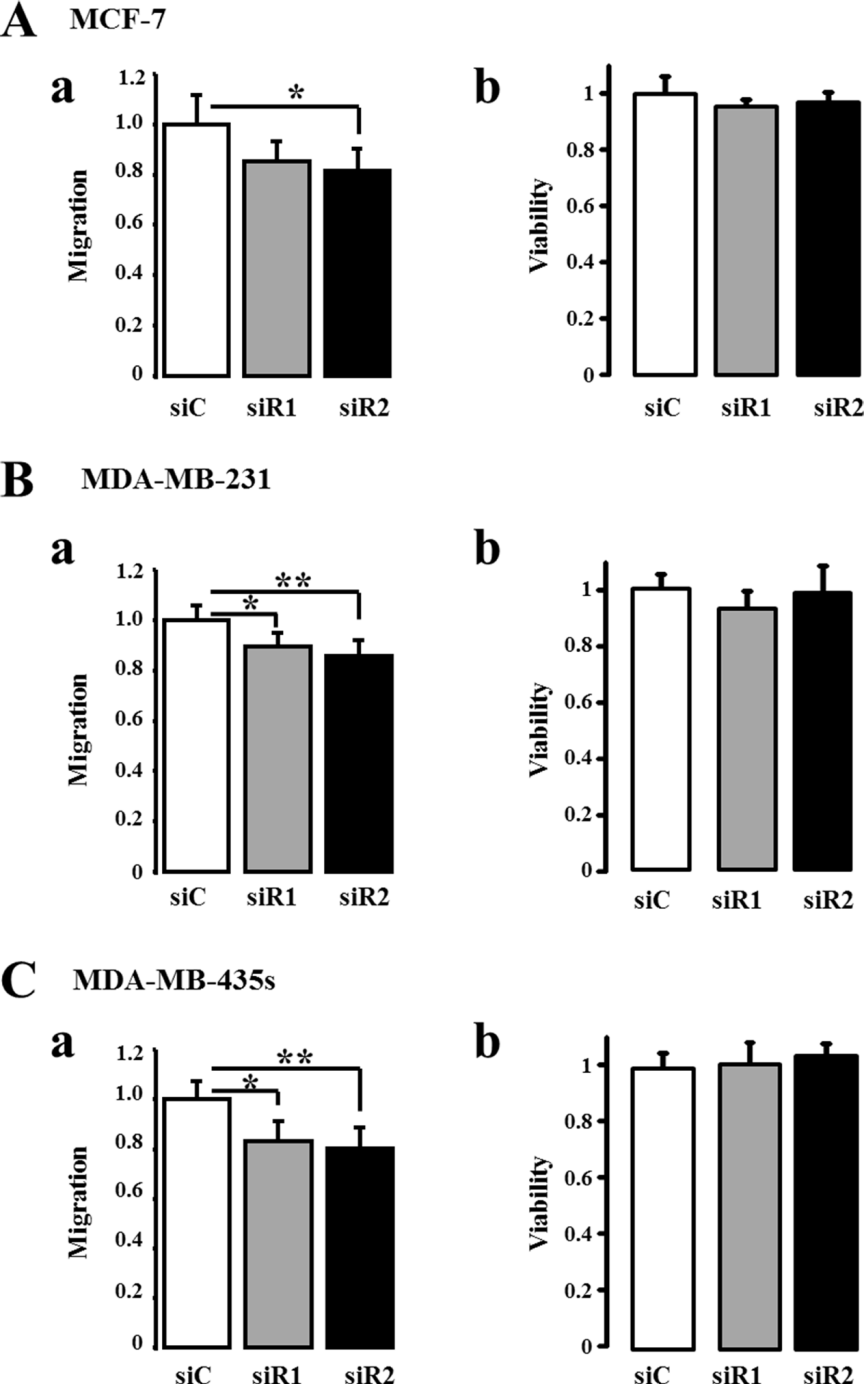
Silencing IP_3_R1 or IP_3_R2 reduce breast cancer cell migration to a lesser extent than IP_3_R3 The three cell lines were transfected either by a small interfering RNA (siRNA) targeting IP_3_R1 (siR1), IP_3_R2 (siR2) or a control siRNA (siC). The migration was then measured at 24 h for MCF-7 (**Aa**) and 18 h for MDA-MB-231 (**Ba**) and MDA-MB-435S (**Ca**) cells using Boyden chamber migration assay. In all cases, the measurement was carried out 48 h after the transfection with respective siRNAs. In all three-cell lines, the cell viability was neither affected by siR1 nor by siR2 (**Ab**, **Bb** and **Cb)** for MCF-7, MDA-MB-231 and MDA-MB-435S cells, respectively). Values are reported as mean ± SEM normalized to the corresponding cells transfected with control siRNA (siC) (*N* = 3). *p < 0.05, **p < 0.01.

In order to confirm that IP_3_R3 is implicated in cell migration, we realized cell migration assays in cells transfected with two siRNA targeting IP_3_R3 (siR3) compared to cells transfected with a control siRNA (siC). For each cell line, the migration was measured at different time duration considering the different migration rate of each cell line. IP_3_R3 being involved in breast cancer cell proliferation as we already described [17, 23], cell migration was assessed during a time frame which does not exceed 24 h where the cell viability, assessed by MTT, is not yet altered. This allows us to throw off the effect of IP_3_R3 silencing on cell proliferation and to ensure that the observed effects are due solely to the change in migration capacities of breast cancer cells. Cell migration and viability were thus tested 24 h after seeding, in the transwell chamber, for MCF-7 cells and 16 h after seeding for both MDA-MB-231 and MDA-MB-435S cells. In both cases, this time corresponds to 72 h post-transfection with respective siRNA. IP_3_R3 gene silencing reduced cell migration of the three cell lines, with a greater effect in the highly migrating MDA-MB-231 and MDA-MB-435S cells (Figure 3Aa, 3Ba, 3Ca, Table 2 and Supplementary Figure 1). In all conditions, the cell viability was not affected by siR3 (Figure 3Ab, 3Bb, 3Cb, Table 2 and Supplementary Figure 1). Figure 3Ac, 3Bc and 3Cc show the efficiency of siR3 transfection in MCF-7, MDA-MB-231 and MDA-MB-435S cell lines, respectively. IP_3_R2 gene silencing also reduces cell migration of the three cell lines (Figure 4Aa, 4Ba, 4Ca and Table 3), whereas IP_3_R1 silencing inhibits migration of the highly migrating MDA-MB-231 (Figure 4Ba) and MDA-MB-435S cells (Figure 4Ca) but not of MCF-7 (Figure 4Aa, Table 3). The efficiency and specificity of IP_3_R1, IP_3_R2 and IP_3_R3 gene silencing following the transfection by specific siRNAs were confirmed by quantitative real-time PCR (Supplementary Figure 2 and Table 4A) and Western-blot (Supplementary Figure 3 and Table 4B) on the three breast cancer cell lines. Nevertheless, IP_3_R1 or IP_3_R2 silencing remains less invalidating on cell migration compared to IP_3_R3 silencing (Figure 3). Like for siR3 conditions, IP_3_R1 or IP_3_R2 silencing has no effect on cell viability (Figure 4Ab, 4Bb, 4Cb and Table 3).

**Table 2:** IP_3_R3 silencing decreases migration capacities of breast cancer cell lines

Cell type	Migration assays		Viability assays	
	siC	siR3	siC	siR3
MCF-7	1 ± 0.24	0.57 ± 0.13^a^	1 ± 0.09	1.04 ± 0.17
MDA-MB-231	1 ± 0.05	0.53 ± 0.15^b^	1 ± 0.05	0.95 ± 0.14
MDA-MB-435S	1 ± 0.06	0.08 ± 0.17^c^	1 ± 0.05	0.91 ± 0.08

Results presented are mean of triplicate assays (*N* = 3). Significant differences are noted with superscript letters and correspond to *p* values listed below: a = 0.04; b = 0.03 and c = 0.001.

**Table 3:** IP_3_R1 or IP_3_R2 silencing modulates breast cancer cell migration

Cell type	Migration assays			Viability assays		
	siC	siR1	siR2	siC	siR1	siR2
MCF-7	1 ± 0.11	0.85 ± 0.08	0.81 ± 0.08^a^	1 ± 0.11	0.95 ± 0.02	0.97 ± 0.04
MDA-MB-231	1 ± 0.05	0.9 ± 0.05^b^	0.86 ± 0.06^c^	1 ± 0.05	0.93 ± 0.06	0.98 ± 0.01
MDA-MB-435S	1 ± 0.07	0.83 ± 0.07^d^	0.8 ± 0.08^e^	1 ± 0.05	1.01 ± 0.08	1.05 ± 0.04

Results presented are mean of triplicate assays (*N* = 3). Significant differences are noted with superscript letters and correspond to p values listed below: a = 0.02; b = 0.04; c = 0.01; d = 0.02 and e = 0.008.

**Table 4:** Silencing of IP_3_R1, IP_3_R2 or IP_3_R3 specifically reduces gene (A) and protein (B) expression of IP3 corresponding receptor in breast cancer cell lines

A				
Cell type		IP_3_R1	IP_3_R2	IP_3_R3
MCF-7	siC	1 ± 0.04	1 ± 0.03	1 ± 0.05
	siR1	0.315 ± 0.06^a^	1.13 ± 0.13	1.94 ± 0.94
	siR2	0.86 ± 0.27	0.397 ± 0.09^b^	1.58 ± 0.37
	siR3	0.76 ± 0.33	1.24 ± 0.12	0.45 ± 0.03^c^
MDA-MB-231	siC	1 ± 0.03	1 ± 0.05	1 ± 0.06
	siR1	0.307 ± 0.04^d^	1.113 ± 0.22	1.113 ± 0.15
	siR2	1.047 ± 0.18	0.32 ± 0.11^e^	1.407 ± 0.28
	siR3	0.876 ± 0.09	1.18 ± 0.1	0.228 ± 0.05^f^
MDA-MB-435S	siC	1 ± 0.05	1 ± 0.06	1 ± 0.04
	siR1	0.383 ± 0.06^g^	1.38 ± 0.15	1.287 ± 0.16
	siR2	1.14 ± 0.22	0.45 ± 0.02^h^	1.463 ± 0.38
	siR3	0.663 ± 0.17	0.91 ± 0.19	0.167 ± 0.02^i^
**B**				
**Cell type**		**IP_3_R1**	**IP_3_R2**	**IP_3_R3**
MCF-7	siC	1 ± 0.161	1 ± 0.122	1 ± 0.042
	siR1	0.494 ± 0.066^j^	0.927 ± 0.106	0.927 ± 0.057
	siR2	0.983 ± 0.111	0.405 ± 0.077^k^	0.945 ± 0.064
	siR3	0.896 ± 0.211	1.096 ± 0.083	0.205 ± 0.038l
MDA-MB-231	siC	1 ± 0.117	1 ± 0.118	1 ± 0.0037
	siR1	0.411 ± 0.092^m^	0.927 ± 0.097	0.947 ± 0.08^1^
	siR2	0.939 ± 0.048	0.436 ± 0.058^n^	1.049 ± 0.072
	siR3	0.915 ± 0.095	0.964 ± 0.086	0.407 ± 0.029°
MDA-MB-435S	siC	1 ± 0.103	1 ± 0.098	1 ± 0.041
	siR1	0.551 ± 0.084^p^	1.017 ± 0.12	0.939 ± 0.084
	siR2	0.969 ± 0.065	0.436 ± 0.053^q^	0.987 ± 0.086
	siR3	0.985 ± 0.079	1.03 ± 0.092	0.289 ± 0.032^r^

Results presented are mean of triplicate assays (*N* = 3). Significant differences are noted with superscript letters and correspond to *p* values listed below: a = 0.0008; b = 0.0026; c = 0.0001; d = 0.0001; e = 0.003; f = 0.0001; g = 0.0002; h = 0.0013; i = 0.0001; j = 0.01; k = 0.0009; l = 0.001; m = 0.007; n = 0.008; o = 0.01; p = 0.01; q = 0.009 and r = 0.0008.

### ATP-induced maintained Ca^2+^ mobilization is an IP_3_R3-dependent signaling pathway

**Figure 5:**
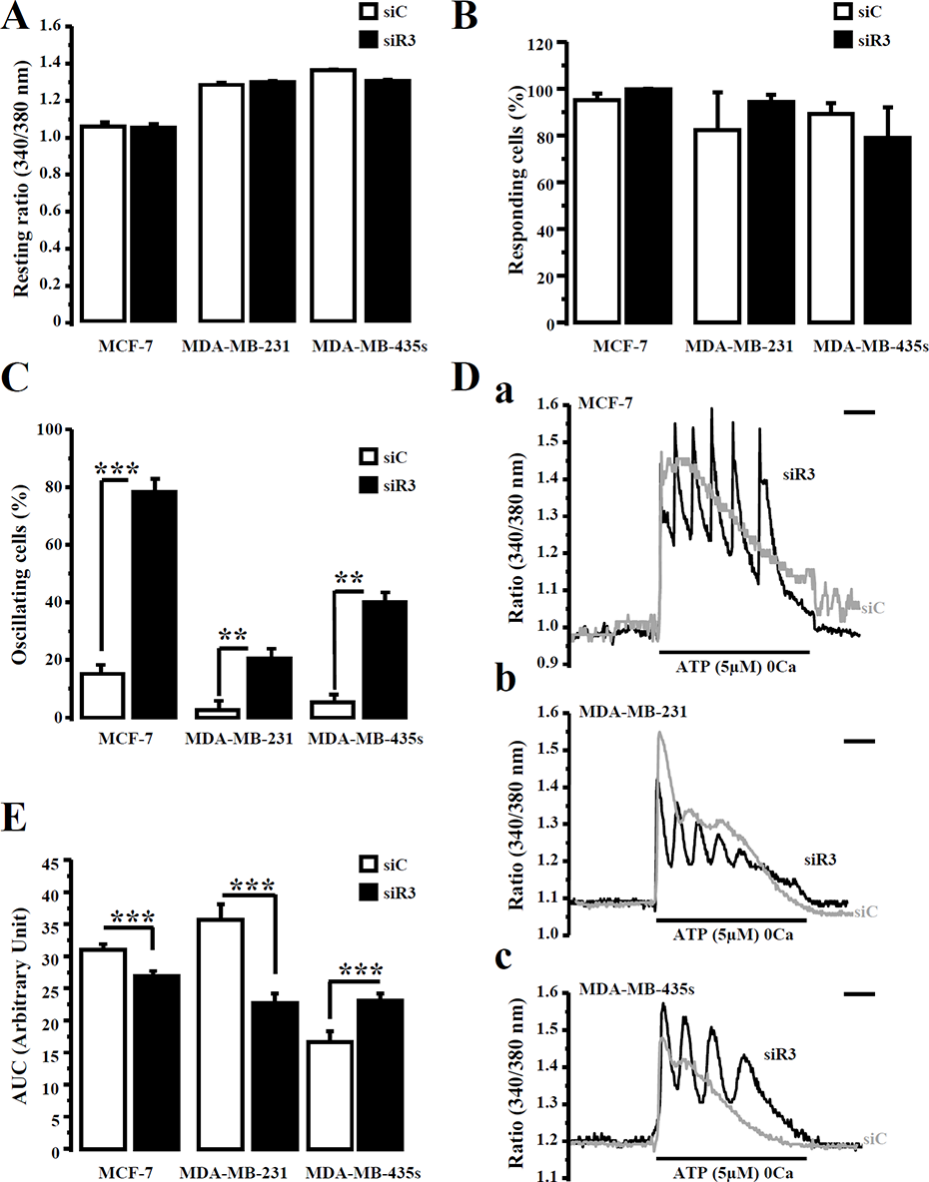
IP_3_R3 silencing reveals an oscillating calcium signal in breast cancer cell lines (**A**) Fluorescence resting ratio (340/380 nm) was measured in Fura-2-loaded breast cancer cells after IP_3_R3 silencing (siR3, black columns) and compared to control cells (siC, white columns). (**B**) Cells number responding to application of ATP (5 μM) in absence of extracellular calcium was compared in the three breast cancer cell lines after IP_3_R3 silencing (black columns) *vs* control transfected cells (white columns). (**C**) In each cell lines, the number of ATP-induced oscillating responses was determined following IP_3_R3 silencing (black columns) *vs* control (white columns) transfected cells. (**D**) Representative ATP-induced Ca^2+^ responses in MCF-7 (Da), MDA-MB-231 (Db) and MDA-MB-435S cells (Dc) illustrate oscillating signal revealed by IP_3_R3 silencing (black traces) compare to control (grey traces) conditions. (**E**) The magnitude of the ATP-induced Ca^2+^ signal was quantified in each cell line after IP_3_R3 silencing (black columns) compared to control transfected cells siC (white columns). Scale bar = 120 s. Values are reported as mean ± SEM (*n* = 73 to 447 cells). **p < 0.01, ***p < 0.001.

**Figure 6:**
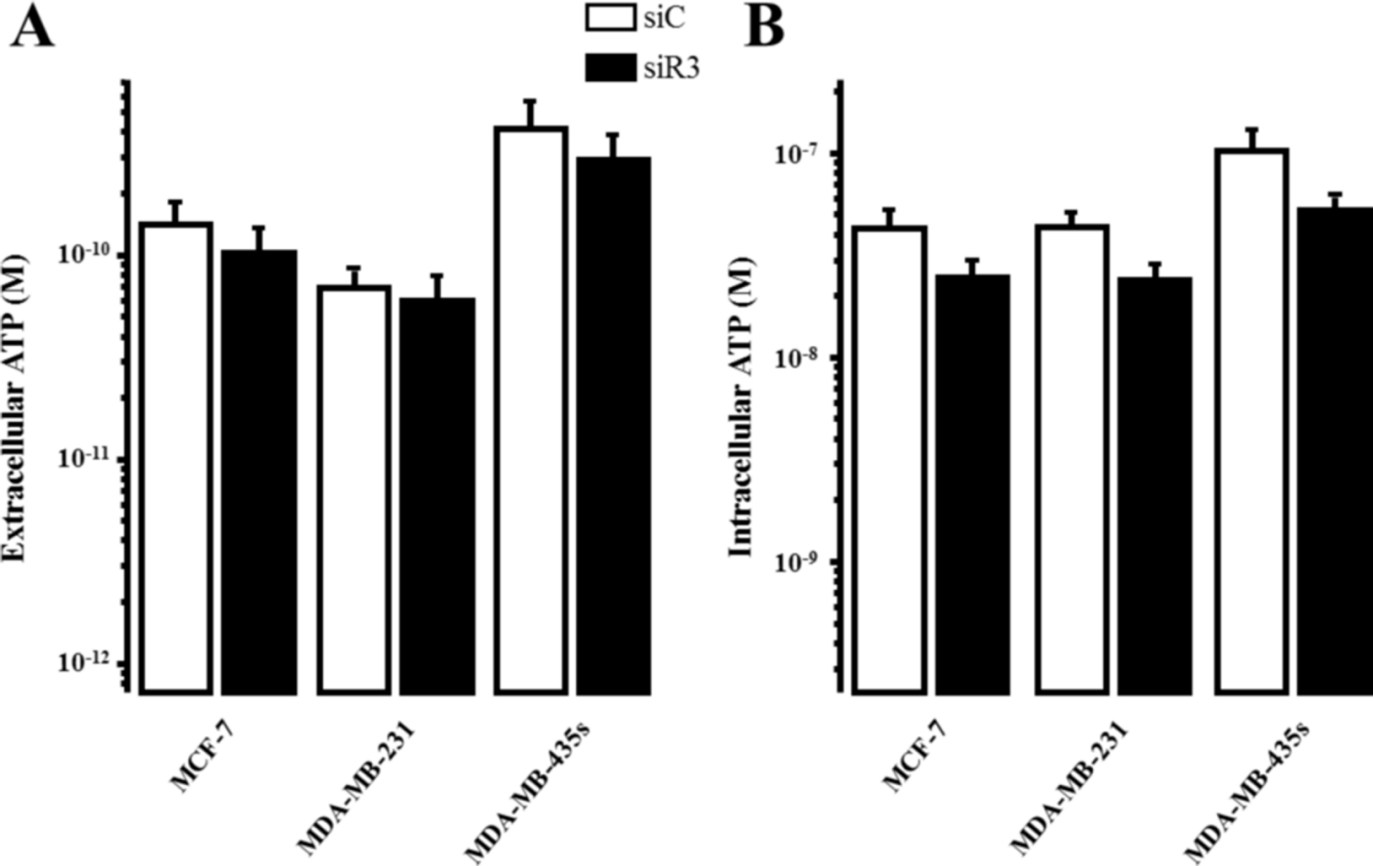
IP_3_R3 silencing has no impact on extracellular- or intracellular-ATP concentrations in breast cancer cell lines (**A**) Extracellular ATP concentration (M) was measured in supernatant, incubated with luciferin/luciferase, of breast cancer cells after IP_3_R3 silencing (siR3, black columns) and compared to control cells (siC, white columns). (**B**) Intracellular ATP released of breast cancer cells transfected with siR3 (black columns) was measured, after incubation of cells with ATP releasing reagent and luciferin/luciferase, and compared to control cells (siC, white columns). Values are reported as mean ± SEM (*N* = 2).

**Figure 7:**
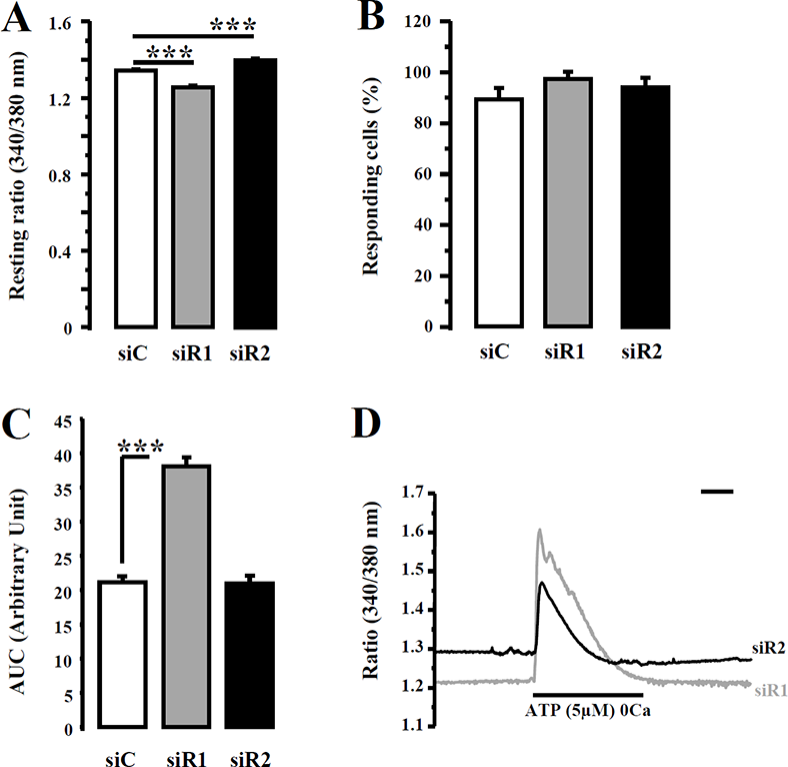
IP_3_R1 or IP_3_R2 silencing has no impact on ATP-induced Ca^2+^ profile in MDA-MB-435S cells (**A**) IP_3_R1 (grey columns) or IP_3_R2 (black columns) expression was reduced using specific siRNAs and the fluorescence resting ratio (340/380 nm) was measured. (**B**) The number of the oscillating cells determined following IP_3_R1 and IP_3_R2 silencing (black columns) *vs* control transfected MDA-MB-435S cells (white columns). (**C**) The magnitude of ATP-induced Ca^2+^ response was evaluated by measuring the AUC after silencing of IP_3_R1 (grey column) or IP_3_R2 (black column). Typical ATP-induced Ca^2+^ response in siR1 (grey trace) or siR2 (black trace) MDA-MB-435S cells both present a transient profile without oscillating component (**D**). Scale bar = 120 s. Values are reported as mean ± SEM (*n* = 129 to 447 cells). ***p < 0.001.

Migration processes implying Ca^2+^ flux [24–26], we investigated whether siR3-induced migration inhibition was associated with a modification of the Ca^2+^ homeostasis. Breast cancer cell lines transfected with siC or siR3 were loaded with Fura-2/AM (2 μM) for 45 min and ATP (5 μM) was applied, in a Ca^2+^-free solution, to prevent ionotropic/purinergic receptors activation [23, 27]. Statistical analyses revealed that IP_3_R3 silencing do not modify neither basal intracellular Ca^2+^ values (Figure 5A) nor the percentage of responding cells to ATP (Figure 5B) in all cell lines (Table 5). We have previously reported that the decrease of IP_3_R3 expression level changes the Ca^2+^ signal profile from a plateau-type to a sinusoidal oscillatory-shaped signal, which is in favor of a diminution of MCF-7 cell proliferation [17]. In this study, we investigated if such signaling pattern could also be related in highly migrating cells transfected with siR3. Statistical analyses revealed that at 72 h post-transfection, the number of oscillating cells in response to ATP is much higher in siR3-transfected cells compared to siC-transfected cells (Figure 5C) in all cell lines (Table 5). Figure 5D represents typical Ca^2+^ signals measured at 72 h post-transfection after perfusion with 5 μM ATP in a Ca^2+−^free medium. In order to determine if this IP_3_R3 signaling profile modification is associated with a change of ATP-induced Ca^2+^ mobilization, we measured the “area under curve” (AUC) for each trace in each cellular condition. Thus, the mean AUC values for Ca^2+^ signals elicited in siC-transfected versus siR3-transfected cells are statistically different (Figure 5E) in all cell lines (Table 5). The AUC values were decreased in MCF-7 and MDA-MB-231 siR3-transfected cells, whereas it increased in MDA-MB-435S siR3 transfected cells. Similar data were obtained with the ΔR/R ratio (Supplementary Figure 4A) except for MCF-7 cells. This discrepancy of AUC values between MCF-7, MDA-MB-231 *vs* MDA-MB-435S cells could not be the consequence of siR3 induced modifications of extracellular (Figure 6A) or intracellular (Figure 6B) ATP basal values as no significant differences could be detected for each condition. Moreover ATP induced-Ca^2+^ oscillations are specific to IP_3_R3 silencing since it is not observed in IP_3_R1- or IP_3_R2-silenced cells (Figure 7D). Indeed, IP_3_R1 silencing significantly decreased the resting ratio, whereas IP_3_R2 silencing increased it compared to control conditions (Figure 7A; 1.34 ± 0.005 *vs.* 1.26 ± 0.007 (*p* = 1.10^−17^) and 1.40 ± 0.01 (*p* = 3.10^−6^) for siC, siR1 and siR2 conditions respectively, *N* = 160 and 129 cells respectively). Silencing IP_3_R1 or IP_3_R2 does not affect the percentage of responding cells to ATP (5 μM) perfusion (Figure 7B). Interestingly, the global amount of Ca^2+^ released into the cell remained unchanged in siR2 conditions (Figure 7C), but is drastically increased in siR1 conditions (21.16 ± 0.81 *vs.* 38.06 ± 1.21, *n* =160 (*p* = 4.10^−28^) and 21.03 ± 0.86, *n* = 129, for siC, siR1 and siR2 conditions respectively).

**Table 5:** IP_3_R3 silencing modifies ATP-induced calcium signal on breast cancer cell lines

Cell type		Resting ratio	Responding cells (%)	Oscillating cells (%)	AUC	Number of tested cells
MCF-7	siC	1.044 ± 0.02	94.97 ± 2.93	8.4 ± 3.5	34.5 ± 1.01	351
	siR3	1.037 ± 0.02	99.65 ± 0.35	74.2 ± 8.6^a^	30.1 ± 0.82^d^	352
MDA-MB-231	siC	1.267 ± 0.01	82.27 ± 16.16	6.5 ± 2.7	39.8 ± 2.63	73
	siR3	1.280 ± 0.009	94.22 ± 3.22	21.6 ± 5.4^b^	25.4 ± 1.63^e^	85
MDA-MB-435S	siC	1.345 ± 0.005	89.22 ± 4.54	7.3 ± 2.5	18.6 ± 1.83	447
	siR3	1.288 ± 0.007	78.95 ± 12.88	17.2 ± 3.7^c^	25.7 ± 1.3^f^	132

Results presented are mean of numerous assays (*N* = 6–12). Significant differences are noted with superscript letters and *p* values a = 4.10^−4^; b = 0.01; c = 0.009; d = 5.10^−4^; e = 3.10^−6^ and f = 0.001.

### IP_3_R3 overexpression enhances the migration capacity of MCF-7 cell line

**Figure 8:**
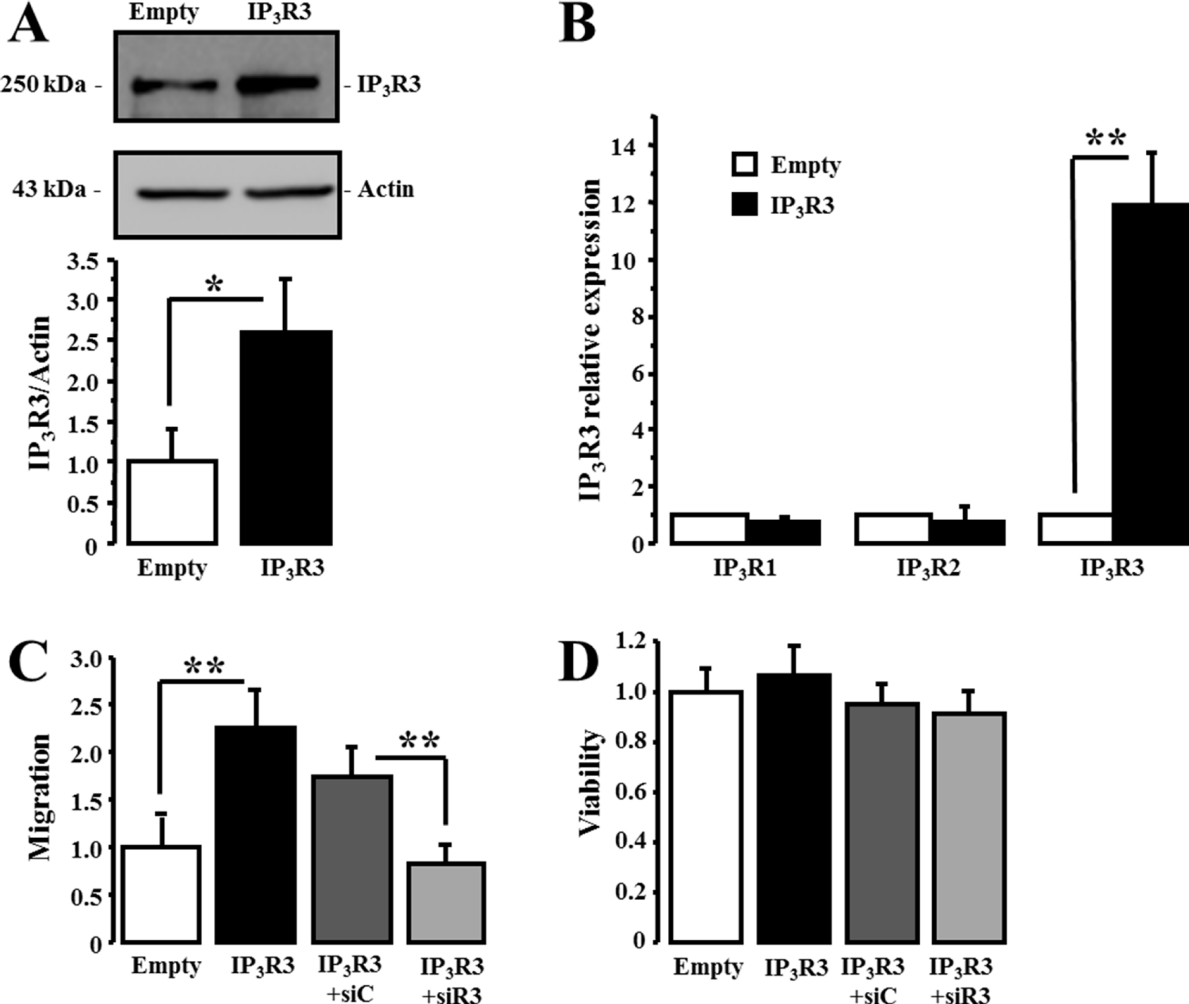
IP_3_R3 overexpression enhances the migration of MCF-7 cells (**A**) MCF-7 cells stably overexpressing IP_3_R3 were generated following transfection with pcDNA(3.1)-IP_3_R3 plasmid. Protein expression level of IP_3_R3 was evaluated by Western-blot (black column) and compared to control conditions (pcDNA(3.1)-empty plasmid) (white column). (**B**) RT-qPCR was carried out to investigate the impact of stable IP_3_R3 overexpression on IP_3_R1 and IP_3_R2 expression levels. (**C**) The effect of stable IP_3_R3 overexpression on the migration capacities of MCF-7 cells was evaluated by Boyden chamber migration assay and the specificity of IP_3_R3-overexpression effect was controlled by an siRNA targeting IP_3_R3 in the pcDNA(3.1)-IP_3_R3 transfected cells. In all experiments, the migration was always measured 72 h after cell transfection with the respective siRNAs and at a time at which there was no effect on cell viability (**D**). Values are reported as mean ± SEM normalized to MCF-7 cells transfected with empty plasmid (*N* = 3). *p < 0.05, **p < 0.01.

**Figure 9:**
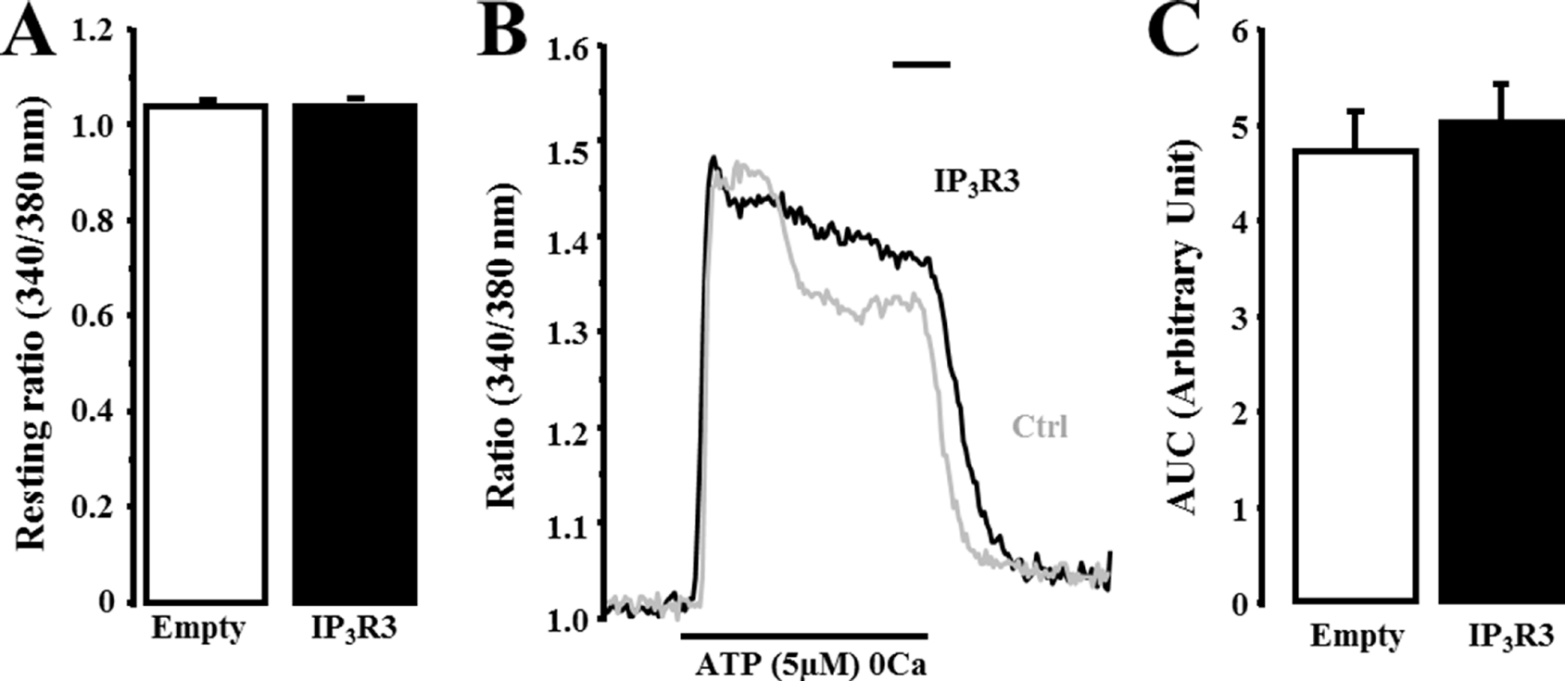
IP_3_R3 overexpression modifies ATP-induced Ca^2+^ transient profile into a sustained Ca^2+^ signal (**A**) Fluorescence resting ratio (340/380 nm) was measured in MCF-7 overexpressing IP_3_R3. (**B**) ATP-induced Ca^2+^ signal was investigated in MCF-7 cells transfected with IP_3_R3 plasmid (B, black trace) compared to the empty plasmid condition (grey trace). (**C**) Magnitude of ATP-induced Ca^2+^ response was evaluated by measuring the area under the curve (AUC) in both IP_3_R3- and empty-plasmid transfected MCF-7 cells. Scale bar = 120 seconds. Values are reported as mean ± SEM (*n* = 32 to 285 cells).

**Figure 10:**
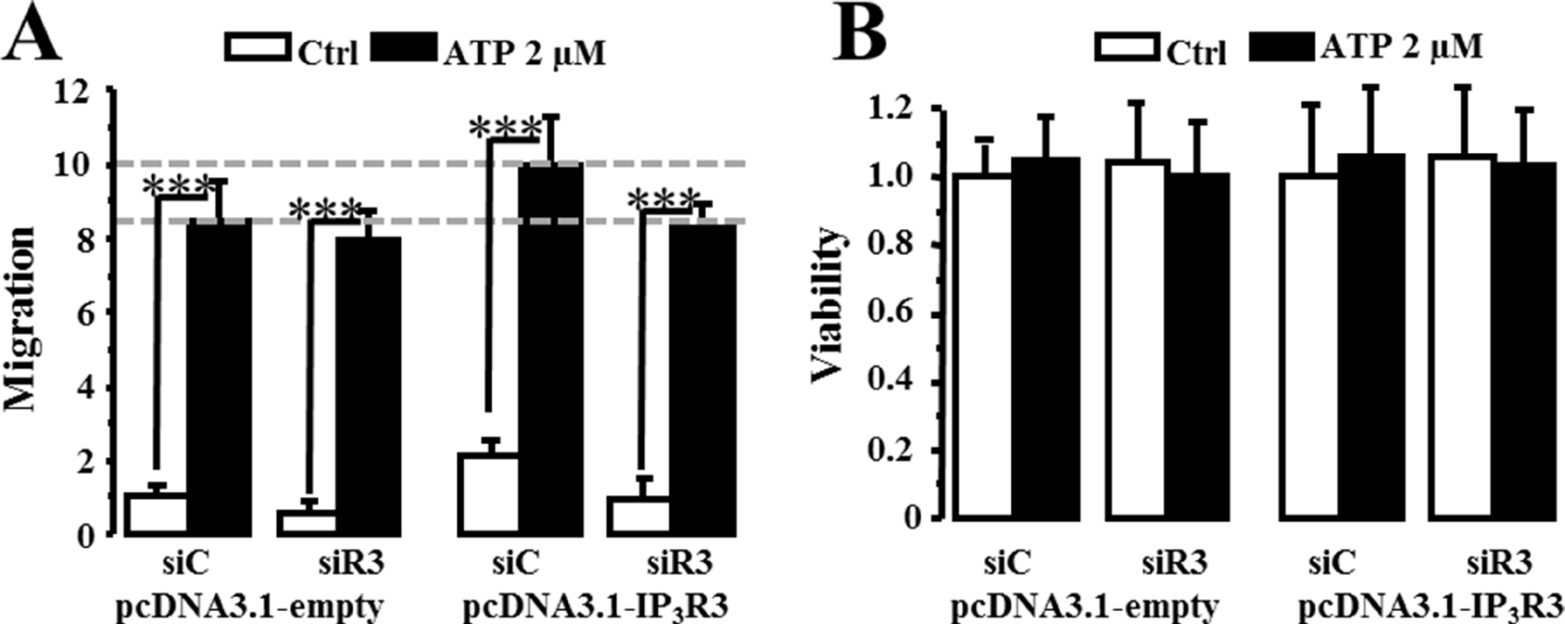
ATP-induced migration is modulated by IP_3_R3 expression (**A**) Migration capacities of MCF-7 stably overexpressing IP_3_R3 were evaluated after incubation with ATP (2 μm) during 24 h (black columns) and compared to non-stimulated cells (white columns). The specificity of IP_3_R3-overexpression effect was controlled by an siRNA targeting IP_3_R3 in the pcDNA(3.1)-IP_3_R3 and pcDNA(3.1)-empty plasmid-transfected cells. (**B**) IP_3_R3 overexpression and ATP (2 μM) stimulation impact on MCF-7 cells viability is controlled by MTT assay. Values are reported as mean ± SEM normalized to the MCF-7 cells transfected with empty plasmid and control siRNA (siC pcDNA3.1-empty) (*N* = 3). ***p < 0.001.

Based on our results showing that siR3 decreased cancer cell migration, we investigated the effect of a stable overexpression of IP_3_R3 in the low migrating MCF7 cell line. The transfection of MCF-7 cells with a pcDNA(3.1) plasmid encoding IP_3_R3 [28] led to a 2.59 fold increase in IP_3_R3 expression level (2.59 ± 0.6 in pcDNA(3.1)/IP_3_R3-transfected cells vs. 1 ± 0.4 in empty pcDNA(3.1)-transfected cells; *N* = 3, *p* = 0.03) (Figure 8A). In order to confirm that the effects recorded were correlated to IP_3_R3 level increase and not to a modification of IP_3_R1 and/or IP_3_R2 expression levels, RT-qPCR analysis of all IP_3_R isoform mRNAs was carried out. Our results show no significant change in the expression of IP_3_R1 and IP_3_R2 at the mRNA level in MCF-7 cells overexpressing IP_3_R3 (Figure 8B). The relative expression level is 0.77 ± 0.17 (*p* = 0.15) of control (empty pcDNA(3.1)-transfected cells) for IP_3_R1 and 0.78 ± 0.51 (*p* = 0.32) of control for IP_3_R2, whereas it is significantly elevated to 11.96 ± 1.81 (*p* = 0.03) of control for IP_3_R3. We then compared migration ability of MCF-7 overexpressing IP_3_R3 *vs* empty pcDNA(3.1)-transfected MCF-7 cells (Figure 8C). The relative migration of IP_3_R3 overexpressing MCF-7 cells is enhanced by more than a 2 fold factor (2.26 ± 0.37 vs. 1 ± 0.33; *N* = 3; *p* = 0.008). Furthermore, the migration of MCF-7 stably overexpressing IP_3_R3 is reduced to control values by siR3 (0.82 ± 0.19; *N* = 3; *p* = 0.01; IP_3_R3+siR3). This effect on cell migration is independent of any effect on cell viability (Figure 8D). We also record intracellular calcium variations in these cells. IP_3_R3 overexpression does not modify Ca^2+^ homeostasis in these cells (1.04 ± 0.001 *vs.* 1.04 ± 0.001 in empty and IP_3_R3 transfected cells respectively, Figure 9A). The perfusion of ATP (5 μM) induced a plateau-type signal that appears more sustained in IP_3_R3 overexpressing cells (Figure 9B) without modify the amount of Ca^2+^mobilized (Figure 9C; 5.02 ± 0.37 *vs.* 4.72 ± 0.39 in empty and IP_3_R3 transfected cells respectively). Indeed, the measure of ΔR/R ratio to Thapsigargin (TG)- or ATP-induced Ca^2+^ response shows no significant differences (Supplementary Figure 4B and 4C). This result indicates that IP_3_R3 overexpression modify rather intracellular Ca^2+^ availability than intracellular Ca^2+^ homeostasis. This Ca^2+^ mobilization maintained during ATP application stimulates migration capacities of MCF-7 cells (Figure 10A). Indeed, migration of MCF-7 cells transfected with the pcDNA(3.1)-empty plasmid is increased following ATP stimulation (2 μM) (8.41 ± 1.2 in ATP treated cells *vs.* 1 ± 0.24 in Ctrl; *N* = 3, *p* = 0.0001). This increase seems to occur independently of IP_3_R3 since IP_3_R3 silencing failed to decrease the ATP-stimulated migration. However, in MCF-7 stably overexpressing IP_3_R3, and was not correlated to a proliferative effect (Figure 10B). ATP strongly increases cell migration (10 ± 1.24 in ATP treated cells *vs.* 1 ± 0.24 in Ctrl; *N* = 3, *p* = 0.03) that is abolished following IP_3_R3 silencing.

## DISCUSSION

It is well known that Ca^2+^ ions, together with many other Ca^2+^-dependent proteins and signaling pathways, play a crucial role in regulating cell migration [25, 26, 29, 30]. For example, capacitive Ca^2+^ entry has been shown to be involved in the migration of breast cancer cells [31], hepatocarcinoma cells [32] and in vascular smooth muscle cells [33]. Conversely, few studies have shown the role of the secretory pathway of Ca^2+^ in cancer cell migration. Thereby RyRs have been found to control astrocyte cell migration [34] whereas the SPCA1 Ca^2+^-ATPase has been shown to be involved in cell migration during *Caenorhabditis elegans* embryonic development [35]. In this context, and based on previous works showing a role of Ca^2+^ and IP_3_R Ca^2+^ release channels in cell motility, we have studied the potential implication of IP_3_R3 in cell migration process in three different breast cancer cell lines showing distinct migration capacities. Our study demonstrates, for the first time, that IP_3_R3 by modifying the calcium signal profile can regulate different breast cancer cell migration capacities. Indeed we establish that (i) MCF-7, MDA-MB-231 and MDA-MB-435S breast cancer cell lines express different levels of IP_3_R3 protein, (ii) cells expressing IP_3_R3 in a larger amount migrate more extensively than the others, (iii) silencing of IP_3_R3 changes a sustained ATP-induced Ca^2+^ increase to an oscillatory one, (iv) overexpression of IP_3_R3 increases the migration capacities of the non-invasive MCF-7 cell line and switch the ATP-induced transient Ca^2+^ response to a sustained phase.

Interestingly, we were able to discriminate a modulation of ATP-induced Ca^2+^ response specifically correlated to IP_3_R3 expression. Indeed, down-regulation of IP_3_R3 in high migrating cell lines reveals an oscillatory ATP-induced Ca^2+^ signal, whereas overexpression of IP_3_R3 in low migrating breast cancer cell line modify the Ca^2+^ signal in a sustained one. These results are in agreement with previous studies showing that IP_3_R3 forms an anti-oscillating unit, its down-regulation revealing a Ca^2+^ oscillating profile specific to IP_3_R2 activation [15]. Interestingly, Ca^2+^ oscillations associated to IP_3_R3 silencing were associated to a reduction of the intracellular Ca^2+^ concentrations in MCF-7 and MDA-MB-231, and an increase in MDA-MB435s cells. Thus, even if IP_3_R3 silencing systematically induces an oscillating Ca^2+^ signature in breast cancer cells, the Ca^2+^ amount involved in this response differ between the various cell lines. This discrepancy could be explained by differences in the expression levels of the purinergic receptors P2 [36–39] or activation of P1 adenosine receptors due to the ATP hydrolysis by ectonucleotidases such as CD39 or CD73 [40]. Moreover a variation of intracellular Ca^2+^ availability through the Mitochondrial Calcium Uniport (MCU) activity [41] could also convey such difference between MCF-7 and MDA-MB-231 *vs* MDA-435s cells.

One of the major issues of the present work lies in the identification, for the first time, that IP_3_R3, by regulating the Ca^2+^ homeostasis in an anti-oscillating profile, impact the migratory capacities of breast cancer cells. Our results are thus supported by other studies showing that IP_3_R3 is overexpressed in various cancer tissues. Indeed, IP_3_R3 expression is found to be increased in gastric carcinoma [21] and glioblastoma [20] where it seems to be involved respectively in peritoneal dissemination and in brain invasion. Kang *et al*. [20] elegantly demonstrate that caffeine, at concentration that inhibits IP_3_R3 preferentially to the two other IP_3_R subtypes, is able to inhibit migration and invasion of glioblastoma cells *in vitro*. Moreover, IP_3_Rs inhibition markedly reduces the migration of pancreatic adenocarcinoma cells by modulating the Ca^2+^ signaling complexes [42]. We establish a link between an oscillating IP_3_- dependent Ca^2+^ signal and the migration capacity of breast cancer cells. Indeed, if Ca^2+^ oscillating signature has been described to modulate dendritic [43] or astrocytoma [44, 45] cell migration; such correlation has never been characterized on cancer cells. These intracellular Ca^2+^ fluctuations, by modulating the availability of free Ca^2+^, may regulate migration processes. A variety of intracellular signaling molecules and their associated biochemical pathways have been identified in the regulation of cell migration such as actin and myosin complexes [46, 47], and Rho subfamily of small G proteins (Rho, Rac, and Cdc42) [48, 49]. This Ca^2+^ signaling has also been shown to activate myosin light chain kinase (MLCK) and control the local disassembly of focal adhesions [24, 50–54]. IP_3_R3-dependent Ca^2+^ oscillations may be the consequences of binding partners like IRBIT [55], Ca^2+^ binding proteins [56], or a close interaction between IP_3_Rs and the Mitochondrial Calcium Uniport (MCU) [57, 58]. Further studies are needed to determine the implication of these actors in IP_3_Rs dependent oscillations genesis.

Altogether, our results clearly indicate that IP_3_R3 is involved in the migration of human breast cancer cells through a specific calcium signature. Overexpression of IP_3_R3 increases cell migration capacities by inducing a sustained Ca^2+^ signature, whereas down-regulation of IP_3_R3 expression reveals an oscillating Ca^2+^ signature along with a slowing down of cell migration. Thus, this work led us to put forward the hypothesis that IP_3_R3, by remodeling the Ca^2+^ signal, is a key player in the migration of human breast cancer cells.

## MATERIALS AND METHODS

### Cell culture

Human breast epithelial cancer cell lines MCF-7, MDA-MB-231 and MDA-MB-435S were obtained from the ATCC (American Type Culture Collection). Cell lines were cultured in the Minimum Essential Medium (MEM, Gibco, LifeSciences, Cergy Pontoise, France) complemented with 0.45% of sodium bicarbonate, 0.06% Hepes, 0.1% of non-essential amino acids, 2 mM L-glutamine, and 5% fetal calf serum (FCS, Lonza, Aubergenville, France). Cell lines were maintained at 37°C and 5% CO_2_ in a humidified atmosphere. Cells were used up to 20 passages after ATCC vial defrosting.

### Cell migration

Migration tests were performed in 8 μm pore sized membrane Boyden chamber (BD FALCON^TM^ Cell Culture Inserts, BD Biosciences, Le Pont de Claix, France) according to the manufacturer's protocol. The upper compartment was seeded with 4.10^4^ cells 48 h after transfection (siC and siR3 or pcDNA(3.1) empty-plasmid and pcDNA(3.1) IP_3_R3-plasmid) in MEM medium supplemented with 5% FCS. The lower compartment was filled with MEM medium supplemented with 5% FCS. After 16 h of further incubation at 37°C for MDA-MB-231 and MDA-MB-435S cells and 24 h for the low migrating MCF-7 cells (as previously described [59, 60]), migrated cells which have passed to the lower side were washed by PBS, fixed in ice-cold methanol and stained by hematoxylin. The remaining cells were removed from the upper side of the membrane by scrubbing. Migrated cells were counted under an inverted microscope (Nikon eclipse TS100 microscope, Champigny-sur-Marne, France) in duplicate (20 contiguous areas at 400 × magnification for each insert). For each experiment, the number of migrating cells per area for each condition (siC and siR3 or pcDNA(3.1) empty-plasmid and pcDNA(3.1) IP_3_R3-plasmid) was normalized to control (siC and pcDNA(3.1) empty-plasmid). For each experiment, a cell viability test was carried out in the same condition than the migration assays.

### Total RNA isolation and quantitative RT-qPCR

After transfection, 5.10^5^ cells were seeded in 100 mm petri dishes in MEM medium 5% FCS. Total RNA was harvested from the cells with the standard trizol-phenol-choloroform protocol. Complementary DNA was synthesized from 2 μg of RNA with random hexamers and MultiScribe^™^ Reverse Transcriptase (Applied Biosystems, Carlsbad, CA). Relative abundance of mRNA was quantified based on Ct difference on a LightCycler real-time PCR machine (Roche, Basel, Switzerland) using a mix containing SYBR green, Taq polymerase and specific primers (Table 6). Results were expressed as gene expression normalized to β-actin expression.

**Table 6:** Primers sequences used for RT-PCR experiments

Primer	Sequence
IP_3_R1 forward	5′-CAGGTTCAACTGCTGGTTAC-3′
IP_3_R1 reverse	5′-CCTTCTCATAGGGAATCTGC −3′
IP_3_R2 forward	5′-AAGCAGGTGCAATTACTGG-3′
IP_3_R2 reverse	5′-CAAAGTGTTGTACAACTCTCTCG-3′
IP_3_R3 forward	5′-CACAGCCATCACCATCAAG-3′
IP_3_R3 reverse	5′-GCTGTAGAAGCCGAAGTAG-3′
β-actin forward	5′-CAGAGCAAGAGAGGCATCCT-3′
β-actin reverse	5′-ACGTACATGGCTGGGGTG-3′

### Western-blot

After transfection, 5.10^5^ cells were seeded in 100 mm petri dishes in MEM medium 5% FCS. Western-blots experiments were performed as previously described [23]. After protein transfer, the membranes were incubated with blocking solution (1-3% bovine serum albumin (BSA) in Tris-buffered saline solution containing 0.1% Tween 20) at room temperature for 1 h. Then, membranes were incubated overnight at 4°C with mouse monoclonal anti-IP_3_R1 (1/500, NeuroMab, Davis, CA, USA), goat monoclonal anti-IP_3_R2 (1/250, Santa Cruz, CA, USA) or mouse monoclonal anti-IP_3_R3 (1/500, BD Biosciences, Le Pont de Claix, France) and goat polyclonal anti-actin (1/1000, Santa Cruz, CA, USA) primary antibodies. Membranes were then incubated with respective secondary antibodies (1/2500–1/5000, Santa Cruz, CA, USA), developed using ECL substrate solution (ECL RevelBlot Intense, Cell signaling, Ozyme, Saint Quentin Yvelines, France), exposed with the MF-ChemiBIS (DNR bio-imaging systems, Neve Yamin, Israel) and analyzed using Quantity one software (Biorad, Marnes-la-Coquette, France). For quantification, actin was used as a loading control.

### Cell transfection

Cell transfection was performed using the nucleofection technology according to the Amaxa Biosystems manufacturer's instructions. For the IP_3_R silencing experiments cells were transfected with siRNAs targeting the three IP_3_R isoforms (siR1, 2, 3, ON-TARGET plus, Dharmacon, GE Healthcare Life Sciences, Velizy-Villacoublay, France) or with scrambled siRNA as a control (siC; siGENOME Non-Targeting siRNA, Dharmacon, GE Healthcare Life Sciences, Velizy-Villacoublay, France). MCF-7 cell line was also transfected with plasmid encoding IP_3_R3 (pcDNA(3.1)-IP_3_R3 plasmid, gift from Professor Jan B. Parys [28]) or with the corresponding empty plasmid (pcDNA(3.1)-empty plasmid) for the overexpression experiments. Briefly, 1.10^6^ cells (MDA-MB-231 and −435 s) or 2.10^6^ (MCF-7 cells) were transfected with 2 μg siRNA or pcDNA(3.1) plasmid. After the electroporation, 500 μL of prewarmed MEM medium supplemented with 5% FCS was added and cells were placed at 37°C for 15 min in a CO_2_ incubator. Immediately after transfection, cells were cultured 48 to 72 h for Western-blot, RT-qPCR, cell migration and calcium imaging experiments.

### Cell viability

In parallel to the migration assay, cell viability was evaluated for the different conditions. Briefly, 2.10^4^ cells were seeded in 6-well plates in EMEM medium 5% FCS. After 16 to 24 h (according to the migration time for each cell line), cells were washed with phosphate-buffered saline (PBS) and incubated with a medium containing 0.5 mg/ml of 3-(4,5-dimethylthiazole-2-yl)-2,5-diphenyltetrazolium bromide (MTT, Sigma, Saint-Quentin Fallavier, France) and incubated for 1 h at 37°C. Medium was removed and 800 μL of Dimethyl-sulfoxide (DMSO) was added to solubilize crystals. The optical density of each sample was read on the microplate reader Infinite F200 Pro (TECAN, Lyon, France) at 570 nm.

### Immunofluorescence microscopy

Cells were cultured on coverslips for 72 h. After washing 3 times with PBS, cells were fixed in 3% paraformaldehyde for 10 min at RT. Cells were then washed twice in PBS, blocked with and permeabilized with PBS containing Triton (0.1%) and BSA (5%) for 30 min and incubated overnight with primary antibodies at 4°C. The following day, cells were washed four times in PBS and labeled with an Alexa Fluor 488-labeled goat anti-mouse and a DyLight549-conjugated goat anti-rabbit secondary antibodies for 1 h in the dark at room temperature. After labeling, cells were washed with PBS and incubated with 4′, 6-diamidino-2-phenylindole1% for 1 min. Cells were then washed two more times and mounted onto slides with ProLong^®^ Gold antifade reagent (Life Technologies, Villebon-sur-Yvette, France). Images were acquired with a Zeiss LSM780 confocal microscope (Marly-le-Roi, France) and analyzed with ZEN 2012 software.

### Calcium imaging

After transfection, 7.10^4^ cells were seeded in MEM medium 5% FCS in 35 mm petri dishes on glass cover slips. After 3 days, cells were loaded for 45 min with Fura-2/AM (2 μM in medium solution) at 37°C in a CO_2_ incubator and subsequently washed with MEM medium. The cover slip was then transferred into a perfusion chamber of a fluorescence Zeiss inverted microscope (Marly-le-Roi, France). Fluorescence was excited at 340 and 380 nm alternately, using a monochromator (Polychrome IV; TILL Photonics, Planegg, Germany), and captured by a Cool SNAP HQ camera (Princeton Instruments, Evry, France) after filtration through a long-pass filter (510 nm). Background fluorescence was determined at 340 and 380 nm from an area of the cover slip free of cells. These values were routinely subtracted. Metafluor software (version 7.1.7.0, Molecular Devices, St. Grégoire, France) was used for acquisition and analysis. All recordings were carried out at room temperature. Cells were continuously perfused with the saline solution, and chemicals were added via the perfusion system. The flow rate of the whole-chamber perfusion system was set at 10 mL/min, and the chamber volume was 1 mL. Recording solution had the following composition (in mM): NaCl (145), KCl (5), CaCl_2_ (2), MgCl_2_ (1), glucose (5) and Hepes (10) at pH 7.4 (NaOH). In experiments where Ca^2+^-free solution was used, Ca^2+^ was omitted and EGTA (0.4 mM) was added to the solution.

### ATP measurements

The luciferin/luciferase detection of ATP was performed with the microplate reader Infinite F200 Pro (TECAN, Lyon, France) with ATP bioluminescent somatic cell assay kit (FLASC, Sigma, Saint-Quentin Fallavier, France). Breast cancer cell lines MCF-7, MDA-MB-231 and MDA-MB-435S transfected with siRNA control (siC) or siRNA targeting IP_3_R3 (siR3) were seeded at 2000 cells/well in white 96-well Nunc dishes with clear bottoms 72 hours before ATP measurements. For extracellular ATP measurements, 100μl of supernatant were incubated with the luciferin/luciferase (FLAAM, Sigma) at a final concentration of 0.04%. For intracellular ATP measurements cells were incubated with 100 μl of ATP releasing reagent (FLSAR, Sigma), before incubation with the luciferin/luciferase (FLAAM, Sigma). To determine the amount of ATP released, a calibration curve was constructed using known concentrations of ATP in solution (1, 10, 100, 1000, 10 000, 100 000 pM). Control experiments were performed to eliminate any drug effect on luciferase activity.

### Reagents

All the products were from Sigma (Saint-Quentin Fallavier, France) unless otherwise stated. Final concentrations were obtained by appropriate dilution of stock solutions so that the solvent never exceeded 1/1,000.

### Statistical analysis

All data are expressed as mean ± SEM of at least three independent experiments. *N* refers to the number of experiments repeated and *n* to the number of tested cells. The Student's *t*-test and one-way analysis of variance (ANOVA) with Bonferroni post-hoc analysis were used to group comparison. Statistical significance is indicated in the figures (* *P* < 0.05; ** *P* < 0.01; *** *P* < 0.001).

## SUPPLEMENTARY MATERIALS FIGURES


