# Supplementary material for: Downregulation of type 3 inositol (1,4,5)-trisphosphate receptor decreases breast cancer cell migration through an oscillatory Ca2+ signal
Source: Oncotarget. 2017 Aug 18;8(42):72324–41. doi: 10.18632/oncotarget.20327 (PMC5641133; doi:10.18632/oncotarget.20327)
Supplement: Supplementary file 1 [file oncotarget-08-72324-s001.pdf]

Downregulation of type 3 inositol (1,4,5)-trisphosphate receptor decreases breast cancer cell migration through an oscillatory Ca^{2+} signal

SUPPLEMENTARY MATERIALS

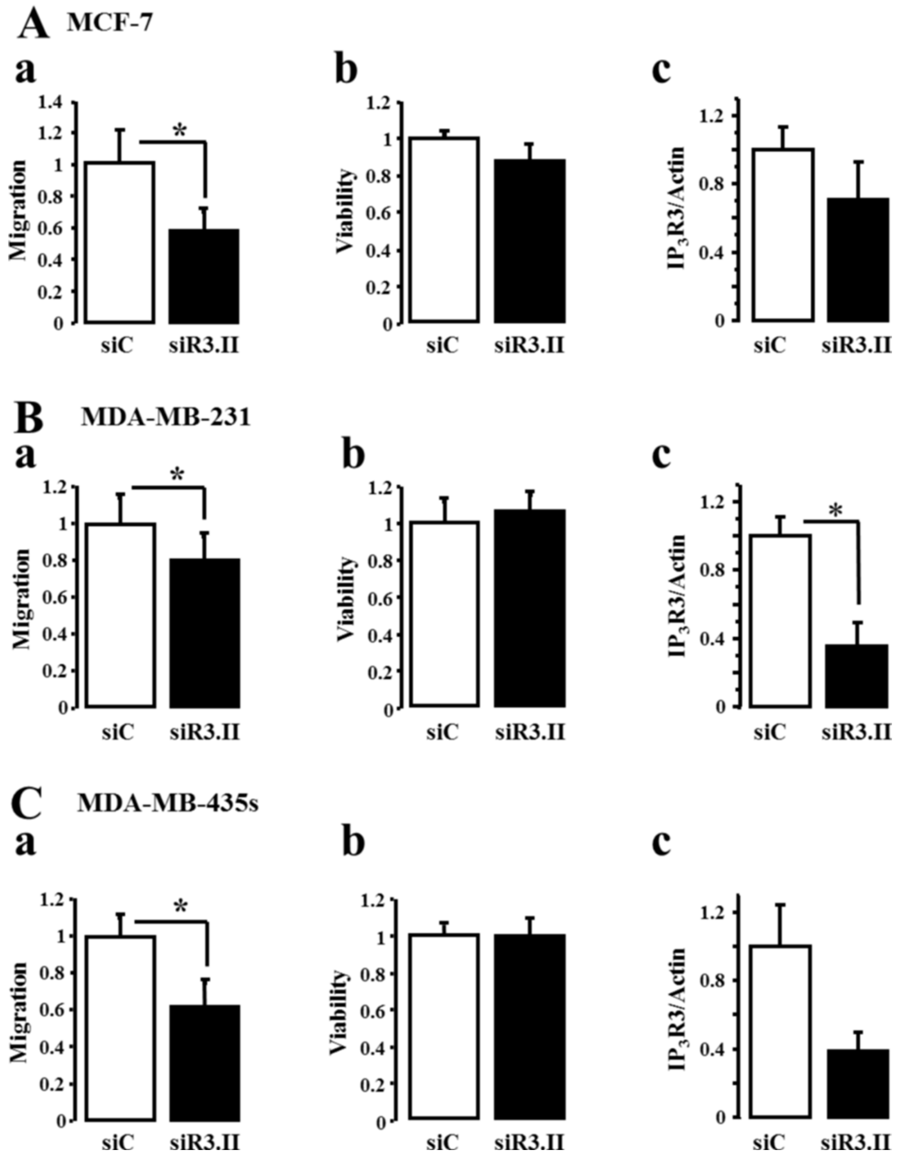

Supplementary Figure 1: Silencing of IP_3R3 inhibits breast cancer cell migration independently of siRNA sequence. The three cell lines were transfected either by a second small interfering RNA (siRNA) directed against IP_3R3 (siR3.II) or a control siRNA (siC). The migration was then measured using Boyden chamber migration assay at 24 h for MCF-7 and 18 h for MDA-MB-231 and MDA-MB-435s cells, according to their different migration capacities and velocities. In all cases, the measurement was made 72 h after the transfection with respective siRNAs. Silencing of IP_3R3 (SiR3.II) significantly reduced migration capacity in the three breast cancer cell lines (MCF-7, (**Aa**) MDA-MB-231, (**Ba**) and MDA-MB-435s cells, (**Ca**) compared to control conditions (siC-transfected cells). In all three cell lines, the migration was measured at a time where cell viability was not affected by siR3.II (**Ab**, **Bb** and **Cb**) for MCF-7, MDA-MB-231 and MDA-MB-435s cells, respectively). Silencing efficiency on IP_3R3 protein expression was confirmed by Western-blot experiments on MCF-7, (**Ac**) MDA-MB-231, (**Bc**) and MDA-MB-435s cells, (**Cc**) Values are reported as mean ± SEM normalized to the corresponding cells transfected with control siRNA (siC) ($N = 3$). *$p < 0.05$.

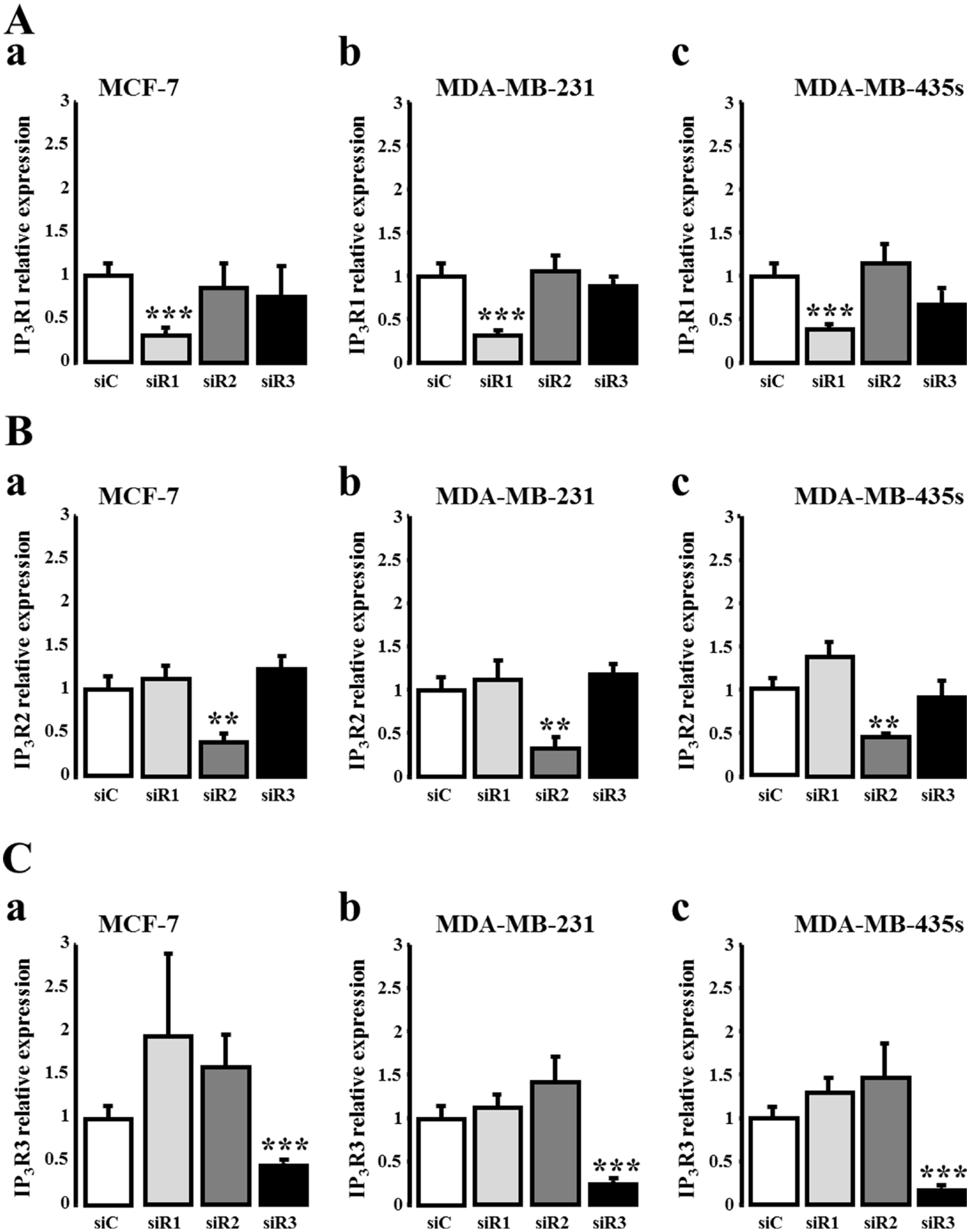

Supplementary Figure 2: Silencing of IP_3R1, IP_3R2 or IP_3R3 reduces gene expression of IP_3 corresponding receptor in breast cancer cell lines. The three cell lines were transfected either by a small interfering RNA (siRNA) targeting IP_3R1 (siR1), IP_3R2 (siR2), IP_3R3 (siR3) or a control siRNA (siC). Specific silencing efficiency on IP_3R1 (**A**) IP_3R2 (**B**) or IP_3R3 (**C**) mRNA expression was confirmed by Q-PCR on MCF-7 (a), MDA-MB-231 (b) and MDA-MB-435s (c) cells. Values are reported as mean ± SEM normalized to the corresponding cells transfected with control siRNA (siC) ($N = 3$). $***p < 0.001$.

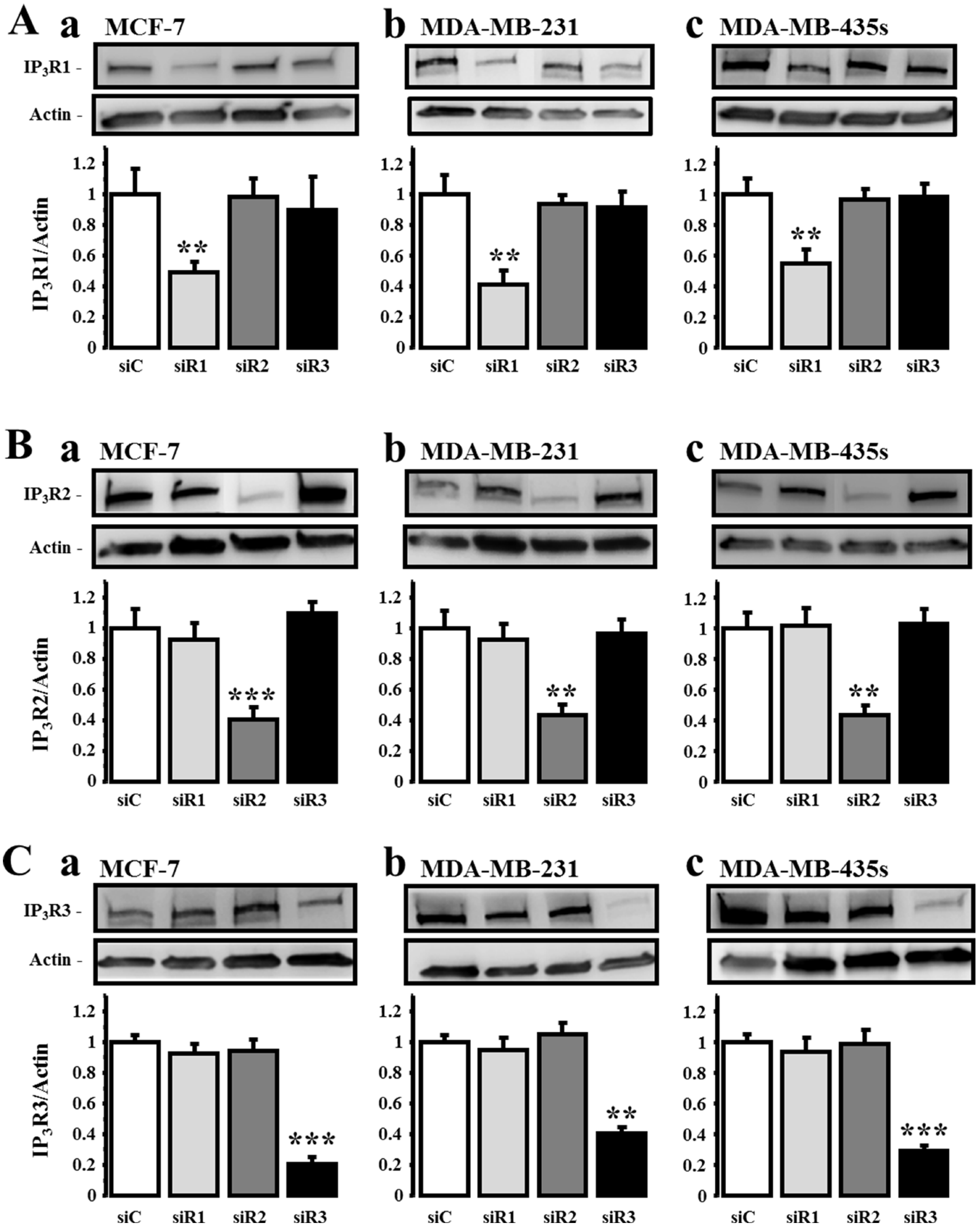

Supplementary Figure 3: IP_3R1, IP_3R2 or IP_3R3 silencing reduces protein expression of their IP_3 corresponding receptor in breast cancer cell lines. The three cell lines were transfected either by a small interfering RNA (siRNA) targeting IP_3R1 (siR1), IP_3R2 (siR2), IP_3R3 (siR3) or a control siRNA (siC). The silencing efficiency on IP_3R1 (**A**), IP_3R2 (**B**) or IP_3R3 (**C**) protein expression was confirmed by Western-blot on MCF-7 (a), MDA-MB-231 (b) and MDA-MB-435s (c) cells. Values are reported as mean ± SEM normalized to the corresponding cells transfected with control siRNA (siC) ($N = 3$). $^{**}p < 0.01$, $^{***}p < 0.001$.

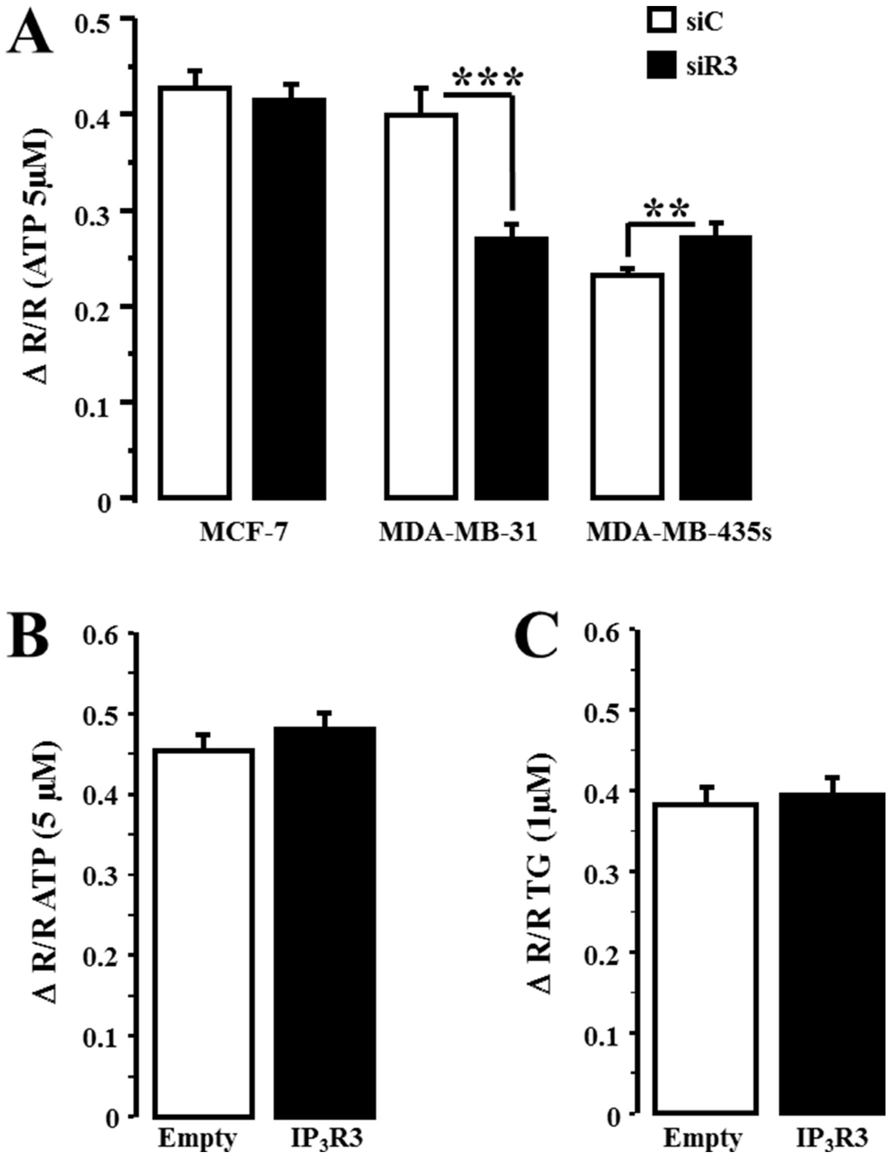

Supplementary Figure 4: Impact of IP_3R3 modulation on ATP- or thapsigargin (TG)-induced Ca^{2+} response. (**A**) silencing IP_3R3 (black column) significantly decreases amplitude of the ATP-induced Ca^{2+} response in MDA-MB-231 cells *vs* siC cells (white column), whereas it is significantly increased in MDA-MB-435S cells. Conversely, overexpression of IP_3R3 in MCF-7 cells neither affects the amplitude of the calcium response induced by ATP- (**B**) nor TG (**C**). Values are reported as mean ± SEM (n = 32 to 447 cells). $^{**}p < 0.01$, $^{***}p < 0.001$.
